# A Simplified and Efficient Method for Himar-1 Transposon Sequencing in Bacteria, Demonstrated by Creation and Analysis of a Saturated Transposon-Mutant Library in Mycobacterium abscessus

**DOI:** 10.1128/mSystems.00976-20

**Published:** 2020-10-20

**Authors:** Mark Foreman, Moran Gershoni, Daniel Barkan

**Affiliations:** a Koret School of Veterinary Medicine, The Robert H. Smith Faculty of Agriculture, Food and Environment, The Hebrew University of Jerusalem, Rehovot, Israel; b Department of Ruminant Science, Institute of Animal Sciences, Agricultural Research Organization, the Volcani Center, Rishon LeZion, Israel; University of Illinois at Chicago

**Keywords:** transposon, genomics, bacterial genetics, *Mycobacterium abscessus*, bioinformatics, mycobacteria, transposon library

## Abstract

We present a technically simple, easy-to-perform method for generating the genomic libraries for Himar-1 transposon site sequencing (Tn-seq). In addition to being simpler than present methods in the technical aspect, it also allows more robust and straightforward identification of the insertion site, by generating a longer sequence surrounding the insertion TA in the genome. The method makes Tn-seq more user-friendly and accessible to laboratories with more-limited bioinformatic resources. Finally, we created a saturated transposon-mutant library in Mycobacterium abscessus and demonstrated the usefulness of the method in analysis of genes involved in colony morphology, as well as in analysis of the whole Tn-mutant library, with identification of over 8,000 unique mutants.

**IMPORTANCE** Transposon insertion sequencing is a powerful tool, but many researchers are discouraged by the apparent technical complexity of preparing the genomic library for deep sequencing and by the complicated computational analysis needed for insertion site identification. Our proposed method makes the preparation of the library easy and straightforward, relying on well-known molecular biology techniques. In addition, the results obtained from the deep sequencing are easily analyzed in terms of transposon insertion site identification, placing library preparation and analysis within the reach of more researchers in the microbiology community, including those with less computational and bioinformatic resources and experience. This is demonstrated by analysis of the most saturated Tn-mutant library created to date in the emerging pathogen Mycobacterium abscessus.

## Abstract (precis)

Transposon insertion sequencing is a powerful tool, but many researchers are discouraged by the apparent technical complexity of preparing the genomic library for deep sequencing and by the complicated computational analysis needed for insertion site identification. Our proposed method makes the preparation of the library easy and straightforward, relying on well-known molecular biology techniques. In addition, the results obtained from the deep sequencing are easily analyzed in terms of transposon insertion site identification, placing library preparation and analysis within the reach of more researchers in the microbiology community, including those with less computational and bioinformatic resources and experience. This is demonstrated by analysis of the most saturated Tn-mutant library created to date in the emerging pathogen Mycobacterium abscessus.

## INTRODUCTION

Random transposon mutagenesis is a powerful tool in bacterial genetics research. Many systems use the Himar-1 (Mariner) transposon, where the only sequence requirement for transposition is a TA dinucleotide sequence. Determination of the exact location of the transposon insertion is labor-intensive, and several methods were developed to accomplish it. One method, highly effective for the Himar-1 transposon, was developed several years ago (1, 2) and is based on introduction of a restriction enzyme (MmeI) recognition site into the inverted repeats (IR) of the transposon, without compromising transposition efficacy. As MmeI digests 20 bp laterally to the recognition site, digesting the genome with it creates a fragment containing the transposon and IR, flanked by 12-bp-long sequences on either side of the TA that was the target of transposition (i.e., the insertion site), with additional two overhang bases. By ligating a mix of 16 different adapters (to accommodate every possible combination of the 2-base overhang) with specialized Illumina-compatible sequences to the overhang bases on either side of the transposon and performing PCR and then Illumina-based deep sequencing, the 14 bp immediately adjacent to the insertion site (16 bp in total—TA plus 14 variable bases) can be identified and used to pinpoint the insertion site. This technique has been successfully used in many transposon library analyses of various bacteria (3–5). However, the technical steps of creating the genomic library for deep sequencing are not simple and necessitate the design of multiple oligonucleotides (to form at least 16 adaptors, and, if tagging is desired, then each tag needs its own 16-bp adaptors) and their hybridization to form the multiple different adaptors to be used as a mixture and are dependent on successful end-to-end ligation of the adaptors to the transposon-containing fragment. Also, the final product of 16 bp (TA plus N_14_) does not always allow unequivocal identification of the insertion site, especially, but not only, in organisms with large genomes and a high number of TA sites.

**FIG 1:**
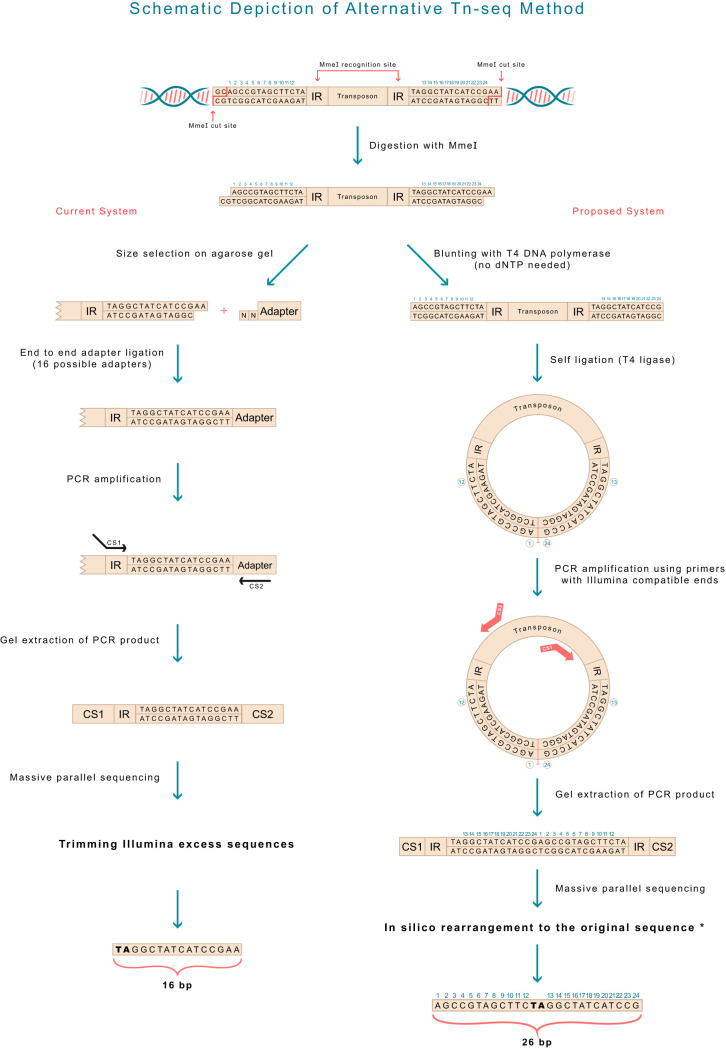
A scheme depicting the technical differences in preparing the genomic library for deep sequencing between the current system (left) and the proposed system (right). Note that the final product in the current system is 16 bp long, whereas in the proposed system, it is 26 bp long. The rearrangement in the order of the bases in the proposed system is shown (bottom right), and the code is presented in Materials and Methods.

Here, we describe a method that simplifies the procedure of genomic library creation, abolishing the need for multiple-adaptor design and the dependence on relatively inefficient end-to-end ligation. In addition, this method allows the identification of a longer, 26-bp sequence (with the insertion of TA in the middle Fig. 1), reducing the number of insertions with equivocal identification by a factor of 1.1 to 7.3 (mean = 2.05) (Table 1), depending on the organism used.

**TABLE 1:** Thirty bacterial organisms were analyzed *in silico* to examine how many Illumina-generated sequences would not be amenable to definitive mapping to the genome (i.e., would have more than one possible mapped location), if the genomic library is prepared in the current system (TA plus N_14_) or the proposed system (N_12_ plus TA plus N_12_), assuming full saturation of the transposon library^*a*^

Organism	Genome length (mbp)	GC content (%)	No. of “TA” within the genome (log_10_)	Total no. of equivocally identified insertions			Accession no.
				Current system (TA + 14N)	Proposed system (12N + TA + 12N)	Ratio	
Clostridium botulinum	4.39	28.02	5.75	141,087	19,134	7.37	NZ_CP013243.1
Clostridium tetani	2.8	28.75	5.54	57,502	13,032	4.41	NC_004557.1
Bacillus anthracis	5.23	35.38	5.66	36,130	9,786	3.69	NC_003997.3
Clostridioides difficile	4.29	29.06	5.71	114,432	36,798	3.11	NC_009089.1
Bacillus cereus	5.42	35.28	5.68	45,823	16,580	2.76	NZ_CP034551.1
Streptomyces avermitilis	9.03	70.72	5.00	12,865	5,442	2.36	NC_003155.5
Sorangium cellulosum	13.03	71.38	5.06	17,638	7,566	2.33	NC_010162.1
Streptomyces coelicolor	8.67	72.12	4.93	13,307	5,934	2.24	NC_003888.3
Staphylococcus aureus	2.82	32.87	5.43	23,371	10,712	2.18	NC_007795.1
Pseudomonas aeruginosa	6.26	66.56	4.97	10,147	5,026	2.02	NC_002516.2
Enterococcus faecalis	3.15	37.23	5.35	14,578	7,342	1.99	NZ_CP041738.1
Chlamydia pneumoniae	1.23	40.58	4.93	1,421	730	1.95	NC_005043.1
Bacteroides fragilis	5.28	43.27	5.54	21,016	11,852	1.77	NC_006347.1
Acinetobacter baumannii	3.98	39.17	5.46	23,498	14,118	1.66	NZ_CP046654.1
Bacillus subtilis	4.22	43.51	5.34	10,574	6,482	1.63	NC_000964.3
Helicobacter pylori	1.68	38.83	5.06	14,117	9,018	1.57	NZ_LS483488.1
Pseudomonas fluorescens	6.52	59.98	5.16	10,783	7,186	1.50	NZ_LS483372.1
Mycobacterium smegmatis	6.99	67.4	4.89	10,530	7,140	1.47	NC_008596.1
Mycoplasma pneumoniae	0.82	40.01	4.75	7,242	4,940	1.47	NC_000912.1
Salmonella enterica	4.81	52.09	5.36	15,433	10,464	1.47	NC_003198.1
Escherichia coli	4.64	50.79	5.33	14,977	10,484	1.43	NC_000913.3
Brucella abortus	2.12	57.16	4.71	3,891	2,868	1.36	NC_007618.1
Azotobacter vinelandii	5.37	65.68	4.92	14,076	10,640	1.32	NC_012560.1
Streptococcus pneumoniae	2.11	39.73	5.14	19,811	15,148	1.31	NZ_LN831051.1
Vibrio cholerae	4.08	47.69	5.28	15,701	12,536	1.25	NZ_CP010812.1
Bartonella henselae	1.93	38.23	5.11	21,120	17,368	1.22	NC_005956.1
Yersinia pestis	4.71	47.7	5.43	35,855	29,474	1.22	NZ_CP033699.1
Chlamydia trachomatis	1.04	41.31	4.84	1,848	1,530	1.21	NC_000117.1
Streptococcus agalactiae	2.28	35.8	5.28	24,794	20,444	1.21	NZ_CP026082.1
Shigella flexneri	4.61	50.89	5.32	42,683	38,414	1.11	AE005674.2

Mean				26,541.67	12,272.93	2.05	

a The factor by which the number of ambiguous sequences is reduced for each organism is shown (“Ratio”) (mean of reduction factors, 2.05).

## RESULTS

After digestion of the target genome by MmeI, rather than running the digest on an agarose gel (see Discussion for other options described in the literature), isolating the gel area with the expected transposon-containing fragment (by size), cleaning it, and ligating to the adaptor mixture, we directly blunt the overhang ends by using T4 DNA polymerase (with 3→5 exonuclease activity). The exonuclease activity is highly efficient, does not necessitate the addition of deoxynucleoside triphosphates (dNTPs), and is done at 12°C for 15 min. The reaction mixture is then cleaned on a DNA purification column, and T4 DNA ligase is added (with the correct buffer) for self-ligation of all the fragments in the mixture (as all would be blunt-ended at that time point). Again, self-ligation is highly efficient even for short incubations at room temperature. Following this, the mixture is cleaned again on a similar column and subjected to PCR with outward-facing primers (referred to here as “outward-looking PCR”) located inside the transposon at anywhere between 50 and 100 bp from the inverted repeat (see Fig. 4). These primers have Illumina compatible ends. Tagging of the primers between the transposon-binding part and the Illumina ends can be done by tagging using 4 or 6 bp. The resulting fragments are 250 to 400 bp long, with Illumina-compatible ends, the first and last 50 to 100 bp of the transposon, both IR, and a 26-bp sequence which is composed of a TA and the two 12-bp sequences (on either side of the transposon). Of note, the primers can be designed to bind closer to or even immediately before the IR, thus making the final product shorter, with possibly better sequencing quality. However, they should not bind the IR itself, as this may result in products that originate from the same primer and that thus have the same Illumina adaptor. After the reads have been obtained from the Illumina procedure, the nucleotides in the sequence (the FASTA files) have to be reorganized to recreate the original order (an easy computational step; Fig. 1, bottom right) and then mapped to the reference genome. As the sequences are composed of 26 bp (N_12_+TA+N_12_) rather than 16 bp, a larger proportion of the sequences can be mapped to their genomic location, with many fewer ambiguous sequences that cannot be traced.

**FIG 2:**
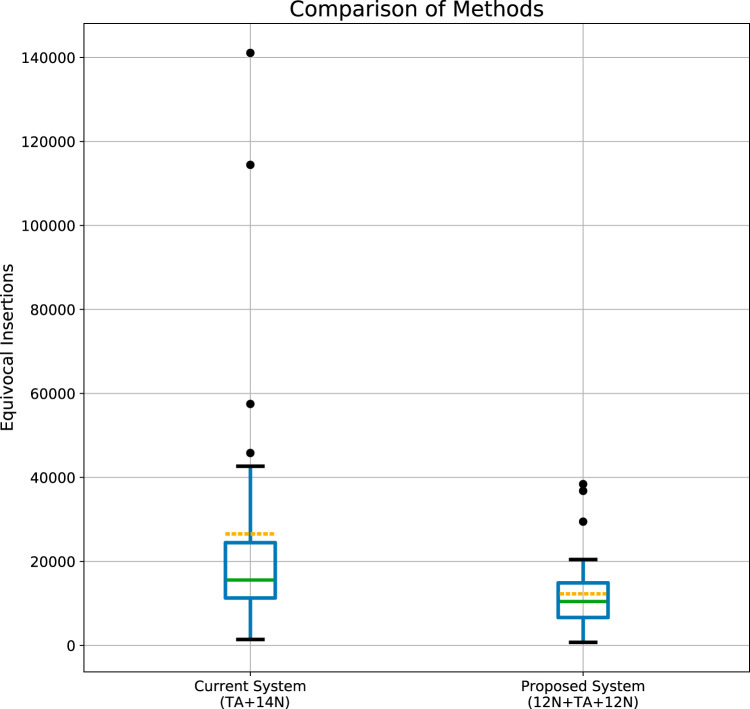
Distribution of equivocally identified insertion sites in the two methods. The number of the equivocally identified insertion sites was calculated for 30 organisms for both methods. The interquartile range (IQR) in the box plot shows the median (green line) and the mean (yellow dashed line). “Whiskers” above the upper quartile and below the lower quartile show the range of the maximum and minimum values. The distribution of the equivocally identified insertion sites is significantly different between the methods; Mann-Whitney U test, *P* = 0.002.

**FIG 3:**
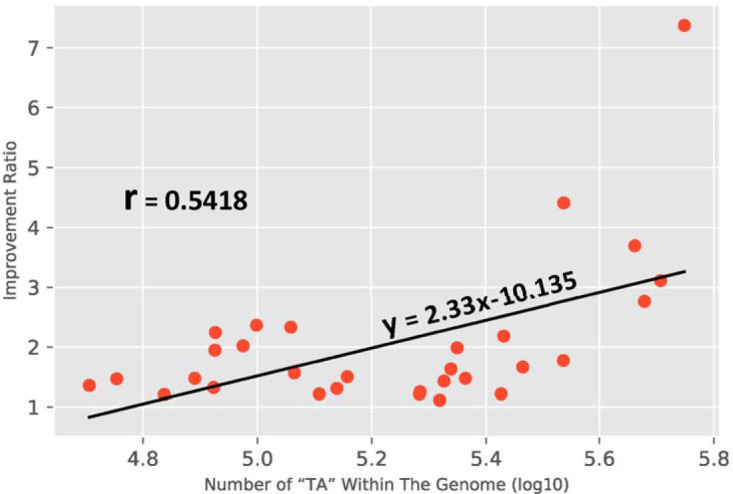
Comparison of the improvement ratio as a function of the number of TA sites. In all analyzed organisms, the proposed system performed better than the current one (the ratio is always above 1), and the degree of improvement was mostly associated with the total number of TA dinucleotides in the genome (*R*^2^ = 0.29).

The technical differences between the present method (Fig. 1, left) and our proposed method (Fig. 1, right) are shown. The *in silico* rearrangement of the 26 bp obtained in the proposed method is also shown. The code details of this rearrangement and of the step of mapping to the genome are available at https://github.com/MarkBio/In-silico-Analysis-Transposon-Sequencing. Table 1 shows the expected improvement in the identification of the Tn site (the reduction in the number of sequences that cannot be equivocally mapped) from the use of the proposed method (generation of 26-bp reads) compared to the present method (with TA plus 14 bp), in 30 different bacteria. The list of bacteria analyzed is composed of several important pathogens, widely used model organisms, and several additional organisms, chosen either randomly or on the basis of unique characteristics such as having the largest known bacterial genome (Sorangium cellulosum). Results of statistical analysis of the improvement in the Tn site identification rate are shown in Fig. 2 (Mann-Whitney U-test, *P* = 0.002), and the improvement is directly correlated with the number of TA sites in the genome (*n* = 30; *r* = 0.5418; *P* = 0.002) (Fig. 3). However, the increase in the number of TA sites explains only about 30% of the variation in the identification rate ratio of our method compared to the present system (*R*^2^ = 0.29, Fig. 3). The improvement in the identification rate is also highly dependent on the abundance of repetitions in the genome and is therefore difficult to predict simply on the basis of GC content, number of TA dinucleotides, or genome length. For example, Salmonella enterica and Shigella flexneri are very similar in genome size, GC%, and number of TAs, but the improvement seen with the proposed system compared to the present one was much more pronounced in S. enterica (×1.47 versus ×1.1). In another example, Streptococcus agalactiae is relatively similar to Staphylococcus aureus in the parameters mentioned above, but the improvement in S. aureus was ×2.18 compared to ×1.2 in S. agalactiae.

### Validation of the proposed method in Mycobacterium abscessus Tn-mutant library.

Specifically, we validated our proposed method by analyzing a M. abscessus ATCC 19977 Himar-1 transposon library (smooth colony morphology), where the antibiotic selection marker in the transposon was zeocin. As the gene conferring zeocin resistance contains an MmeI recognition site, we introduced a silent mutation abolishing the site, with no effect on the amino acid sequence. Previous transposon libraries created in M. abscessus were based on kanamycin selection and used a Himar-1 transposon—albeit without the modification enabling MmeI digestion and high-throughput analysis (6).

**FIG 4:**
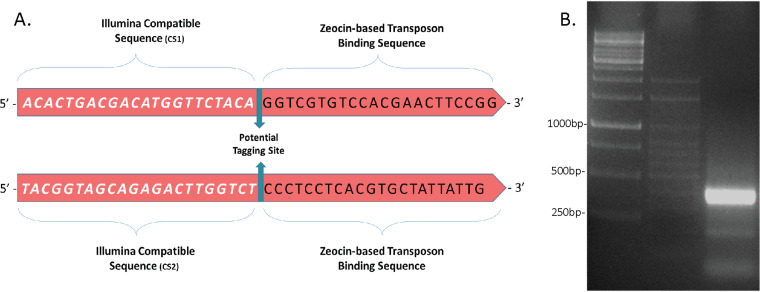
The outward-looking PCR used to create the 400-bp fragments sent for parallel sequencing. (A) The two primers used. If tagging is desired, the area to insert a 4- or 6-bp tag is marked (green arrows). (B) Agarose gel showing the 400-bp product (rightmost lane), next to two DNA ladders.

We first opted to characterize genes where inactivation by the transposon caused an S→R colony morphology transition, as this transition is highly associated with pathogenesis (7). Whereas some of the gene inactivation events responsible for this (such as inactivation of genes encoding the glycopeptidolipid [GPL] complex) are well characterized, others remain unknown (7). We therefore infected M. abscessus ATCC 19977 with a mycobacteriophage carrying our novel transposon (zeocin selection and MmeI-compatible inverted repeats) and picked approximately 200 rough, zeocin-resistant colonies (representing no more than 200 separate transposition events, but probably fewer, due to the same event occurring two or more times). These 200 colonies were pooled, genomic DNA (gDNA) was extracted, and the genomic library was prepared (see Materials and Methods). PCR was performed on the self-ligated fragments with the primers shown in Fig. 4A, and the 400-bp PCR product is shown in Fig. 4B. Illumina-based, mass parallel sequencing was performed using a kit providing a minimum of 250-bp reads. The experiment was repeated four independent times to test for bias and reproducibility, and the details are shown in Table 2. These sequences were mapped to the annotated ATCC 19977 genome, including both the positive and negative strands (https://mycobrowser.epfl.ch/). Each of the sequences was mapped to a single location, with no equivocal Tn site (TA dinucleotide) identification. (Code details are available at https://github.com/MarkBio/In-silico-Analysis-Transposon-Sequencing.) To reduce the incidence of false-positive results, we continued the analysis only for those sequences that came up in both the R1 and R2 analyses. As shown in the table, we found a total of 89 unique TA insertions (79 in coding regions and an additional 10 in noncoding regions). The list of identified genes is shown in Table 3 (for interrupted coding regions) and Table 4 (for insertions located in noncoding regions) and represents genes/areas where the transposon's effect was seen as a switch in morphology from smooth to rough, a key step in the pathogenesis of M. abscessus. Whereas some of the affected genes (such as *MAB_4098* and *MAB_4099*) are known to be involved in the S→R transition, others represent novel findings that we intend to explore in the near future.

**TABLE 2:** Transposon-insertion library preparation in M. abscessus and pooling of ∼200 “rough” colony morphology mutants^*a*^

Total no. of sequences obtained from Illumina	No. of unique sequences perfectly fitting the predicted pattern of IR-28 bases-IR (sequenced from R1/R2)	No. of unique TA sites mapped to the genome present in both R1 and R2 readings	Total no. of unique mutants identified from the 4 experiments combined
924,432	1,133/1,084	73	89
1,142,544	2,615/2,377	65	
1,052,922	3,569/3,090	71	
1,191,602	3,536/2,923	77	

a The analysis of 200 rough colony morphology clones, isolated from a transposon-mutant library, was repeated four independent times. The procedure used was as follows: (i) gDNA extraction from pooled rough mutants, (ii) MmeI digestion, (iii) blunting, (iv) self-ligation, and (v) PCR and NGS. The analysis resulted in a total of 89 hits (unique TA insertions). The details of the sequencing and analyses and the combined number of identified insertion sites (representing a disrupted gene or affected region between genes) are shown.

**TABLE 3:** The combined insertion sites found in coding regions in the four separate analyses of a rough-colony M. abscessus transposon-mutant library^*a*^

Hit sequence (12N + TA + 12N)	Locus	Gene length	Insertion point (base within gene)	Strand orientation
CTCCCCGACGAC**TA**CAGCTTTACGCT	MAB_0023	918	711	+
GGGACGGGTACT**TA**TCGTGGGCAGCG	MAB_0062	1,404	548	+
CCCGTCACGGTG**TA**CTGCGGCTCGGG	MAB_0071c	837	693	−
GGGCGCGCAACA**TA**TACCGCTCGGCG	MAB_0121	327	137	+
TGGCGGTCCGTA**TA**ACACCGCGATCG	MAB_0359	927	101	+
ACAACAATCTCC**TA**TACGGGCGATCC	MAB_0434c	1,443	183	−
CCTCACCGTACC**TA**TCACTCCTGCGC	MAB_0495c	423	130	−
CAAGTCCGTATC**TA**TCTACAGAAAGC	MAB_0501c	1,647	999	−
TGGCATGTCGAC**TA**CATGTTCATGCC	MAB_0659	870	297	+
GGCCTGTGGACG**TA**TCTGATGGGCCG	MAB_0677c	972	717	−
ATTTCCAAGGCG**TA**CAGAACTATTCG	MAB_0737c	378	169	−
GTGACCGGCACC**TA**CGACCGCAACTC	MAB_0841	993	576	+
GGGCGGGAGGCC**TA**CGCGGCCAAGCT	MAB_0962	2,403	570	+
TCATTCAGGAGC**TA**CGCGCCAAGCAG	MAB_0962	2,403	259	+
ACCCGGGATCAA**TA**CTGCCCTCGGGC	MAB_0978	2,235	967	+
CTGGCCGAGCCT**TA**CCTCCTGGAGGG	MAB_1003c	819	531	−
ATCCAGTGCTAC**TA**CGGCTTCTACGC	MAB_1011c	858	708	−
ACCGCATCAACT**TA**TGTACCCCCGAT	MAB_1011c	858	15	−
CGAATCGTGGCC**TA**CGGTCGTGTCCG	MAB_1019c	1,071	608	−
GGAAAAGATACC**TA**TAGCCGAGCAGA	MAB_1103	729	369	+
CCCGCTGCCCTT**TA**CTTATCCGCCTT	MAB_1304	1,248	269	+
ACCAATATCTGC**TA**CACCGGCTGCCG	MAB_1319	2,646	1,743	+
TTCATCGCGCTA**TA**CCTGATCGATCA	MAB_1338	1,200	732	+
CGATGGCCTCAA**TA**AGGTCATCGCGG	MAB_1470c	1,446	659	−
CGGTTGACGCAC**TA**CATGGCGCTGAT	MAB_1638	648	417	+
ACTCGTTACTTC**TA**CGAGTGCTTCGA	MAB_1638	648	174	+
GCTACCGGGCTA**TA**CGGCGGACGAAC	MAB_1638	648	27	+
CCTGGGTAGTAA**TA**GCATTCGTCGTC	MAB_1814	348	70	+
TACCTGTATCAC**TA**CACCAGCGCTGC	MAB_1826	1,071	108	+
GAGCTGGCGATG**TA**CACGTTGTTCCT	MAB_1841c	540	285	−
GTCGGCCTGGAA**TA**CGACCCTGAATA	MAB_1851	1,140	159	+
ACCTGCGACGCG**TA**CTGCACGGTGAG	MAB_2054	468	250	+
GGCACCAAGTAC**TA**CATCTCCGGTGT	MAB_2065c	1,155	462	−
GAACAGGAAACA**TA**CATCGATCTCGC	MAB_2174	363	24	+
GATCGCCGCGCA**TA**TAACCCTGGGCG	MAB_2444c	804	533	−
CGGCCTATGCGG**TA**TACGCGCTGCCC	MAB_2514c	765	694	−
GCACGCGCAAGC**TA**CACATTCCCGTC	MAB_2545c	984	436	−
TGCCGCCAAACG**TA**TTGGTGCGAGCC	MAB_2569c	759	251	−
ACGGACCGTCTG**TA**CGACGCGGGGAT	MAB_2975c	1,047	894	−
GGCCGGCGCCGT**TA**AGTTCAAACGCA	MAB_3040c	1,155	14	−
CGTCGGGTCAAG**TA**ACTGTTGATACG	MAB_3049c	420	142	−
ATCACCGCCATG**TA**CGAAATGCACGC	MAB_3073	657	510	+
CCCCATGACTAC**TA**CTGGCGAGCGGG	MAB_3076	1,365	51	+
GCGGCACGGCAA**TA**CGTCCGCAAGAT	MAB_3121c	270	78	−
GATCGGCACTGT**TA**TGTCGCGGTTGC	MAB_3543c	708	599	−
GGGTAACCTACG**TA**CGCTATTGACCG	MAB_3651	4,554	2,003	+
TCGGGGAAGGCA**TA**TGTGCAATGGGG	MAB_3728c	447	414	−
GATCTCCGGTGG**TA**TGCGCAAGCGTG	MAB_3871c	1,149	452	−
TCCAAAGAGCTG**TA**CGAGATCCGCAA	MAB_3871c	1,149	249	−
ATCCAGATCAGT**TA**TGACGACCGCGG	MAB_3934c	837	36	−
CTGCAGGGCTGC**TA**CAAGATCGCCCT	MAB_3972c	1,512	744	−
AATCAGAGTGAA**TA**CAGCCGTAAGCG	MAB_4088c	870	303	−
AATTGGGGGAGA**TA**CAAGCGGCCTTG	MAB_4098c	7,746	1,327	−
CTCATCCGGGTG**TA**CGAGGCGCTGGC	MAB_4098c	7,746	6,702	−
GCGTACATGACC**TA**TACCTCGGGCAC	MAB_4098c	7,746	558	−
ATGCCGCTGATG**TA**CACGGTGGACCT	MAB_4098c	7,746	3,030	−
CATCTATGGGCA**TA**CGCCGTTGCTCA	MAB_4098c	7,746	5,057	−
CGCTCGACGACG**TA**CGGTGAGCTCGA	MAB_4098c	7,746	4,734	−
AAACTCAAGCCA**TA**CACCTACATTTC	MAB_4098c	7,746	6,930	−
CGCTGACACCCA**TA**CAAGAAGGTCTG	MAB_4098c	7,746	3,316	−
CCGTTGCCGATC**TA**CTGGATCAGTTG	MAB_4099c	10,365	5,458	−
ACCATGGACACC**TA**TGCCAGTGCCGG	MAB_4099c	10,365	8,286	−
CGCGTGGACAAT**TA**GATATCTGGCTT	MAB_4099c	10,365	46	−
CGTCTTCCTCCC**TA**TATGGTTCCGGC	MAB_4099c	10,365	2,748	−
CCAAGCCAGGCG**TA**TGGCCGTTGTGC	MAB_4099c	10,365	1,933	−
AACAGGGACTGC**TA**TTCCAGGCCGGA	MAB_4099c	10,365	4,582	−
GTTGGAGAGTTG**TA**TGTGGCCGGCTC	MAB_4099c	10,365	6,936	−
GCCTGCCTGGAG**TA**CATCGAACAAAA	MAB_4100c	231	150	−
GCCGAGCTGCAC**TA**CCTGTTCGAGAA	MAB_4163c	1,647	279	−
ACGAGGGGTACG**TA**CAGGTCGCCGAG	MAB_4207	1,350	316	+
AACATCCTCTAC**TA**CATGGTGGTCAC	MAB_4485	1,347	795	+
GAATCCCGTTGC**TA**TGTACCTGGTTC	MAB_4,554c	1,485	680	−
CTGCTGCGGTTG**TA**CTGGCGGCGGTG	MAB_4573c	918	537	−
GCCGGCGAGCAG**TA**TCTCGACTTCCG	MAB_4594c	1,359	387	−
GGTGCGCGCATG**TA**TCGCACCGGTGA	MAB_4691c	24,327	21,003	−
GCTTGGGTTGGT**TA**TGTGTTCCTCAT	MAB_4803	378	135	+
TGGGGCGTGCTC**TA**CTACGCCTTCCC	MAB_4839c	1,194	102	−
CGACACGGTCAT**TA**CGGTGGGCAAGC	MAB_4867c	1,965	371	−
GCTTAGCCGAAC**TA**CCTTCGGCACTG	MAB_4891c	2,541	1,609	−

a The identified genes are candidates to be involved in an S→R colony morphology transition. The TA hits are indicated in bold.

**TABLE 4:** Loci of hit sequences found in noncoding regions in the four separate analyses of a rough-colony M. abscessus transposon-mutant library^*a*^

Hit sequence (noncoding regions)	Downstream locus	Upstream locus
TAACAGCGGCCT**TA**ATAGGAAAATAG	MAB_1825c	MAB_1826
GCCCGTGGTGTG**TA**CACACCGCGGCT	MAB_0826c	MAB_0827
GCCACAACGGCG**TA**CCAGCATCGCCT	MAB_4857	MAB_4858
GCAAGGGTTACC**TA**TGTTGAGCGGCA	MAB_2750c	MAB_2751
GACCACTCTCGT**TA**GGGGCGTGTGGG	MAB_2796	MAB_2797c
CGCGCCGTCGCT**TA**TTACCCTGTGGG	MAB_0586	MAB_0587
CCGGTTCGTGTG**TA**TAGTGGGGTTAC	MAB_0431c	MAB_0432
CCCTGTGCGTTA**TA**AGTTGAAAGGGG	MAB_3189c	MAB_3190c
CAGGAACTGGCT**TA**ATTGGTCGCTTA	MAB_1992c	MAB_1993
CTCAACAGCATT**TA**CCACGCAGTCAC	MAB_2670	MAB_2671

a The identified genes are candidates to be involved in an S→R colony morphology transition. The TA hits are indicated in bold.

We then continued to perform preliminary analysis of the full mutant library, to demonstrate the effectiveness of the proposed method. The procedure of creation and analysis of Tn-mutant libraries in M. abscessus is notoriously difficult, and the most comprehensive one created to date contained ∼6,000 kanamycin-resistant mutants (6). After pooling all the zeocin-resistant mutants, we performed the procedure twice, with the results shown in Table 5. As seen, the library was found to contain at least 8,008 unique mutants, with at least 2,889 genes (of a total of 4,992 annotated genes in the M. abscessus chromosome) hit by the transposon. This therefore represents the most highly saturated Tn-mutant library in M. abscessus created to date. The preparation of the genome for the sequencing as described here was done within 2 days. The *in silico* analysis was performed as described here, with simple identification of the insertion site and no ambiguously identified sequences. Complete lists of the genes (and noncoding regions) hit and of the exact gene annotation and number of hits per gene are shown in Tables S1 and S2 in the supplemental material.

**TABLE 5:** Saturated transposon-mutant library preparation in M. abscessus ATCC 19977^*a*^

Total no. of sequences obtained from Illumina	No. of unique sequences fitting the predicted pattern of IR-28 bases-IR (sequenced from R1/R2)	No. of unique TA sites mapped to coding regions (no. of affected genes)	No. of unique TA sites mapped to noncoding regions	Total no. of unique mutants identified in the combined 2 experiments
4,880,045	113,256/101,189	5,779 (2,723)	1,473	8,008 unique mutants (representing 2,889 annotated genes)
5,075,842	70,284/62,650	4,205 (2,329)	956	

a The same library analyzed previously only for rough mutants (Table 2) was analyzed for all the resulting mutants. The analysis was done two independent times. The procedure used was as follows: (i) gDNA extraction from pooled rough mutants, (ii) MmeI digestion, (iii) blunting, (iv) self-ligation, and (v) PCR and NGS. A total of 8,008 unique TA sites were hit, representing 2,889 genes (out of 4,992 genes in M. abscessus ATCC 19977). A total of 1,499 TA sites were hit in what are annotated as noncoding regions. A total of 6,508 unique TA sites in 2,889 genes were hit in coding regions.

10.1128/mSystems.00976-20.1 TABLE S1 The table shows all the genomic locations and the gene annotations of the transposon hits in coding regions of the M. abscessus ATCC 19977 chromosome. Altogether, there are 2,889 gene hits, from a total of 4,992 genes in M. abscessus. Download Table S1, XLSX file, 1.4 MB. Copyright © 2020 Foreman et al. 2020 Foreman et al. This content is distributed under the terms of the Creative Commons Attribution 4.0 International license.

10.1128/mSystems.00976-20.2 TABLE S2 The table shows all the genomic locations and the gene annotations of the transposon hits in noncoding regions of the M. abscessus ATCC 19977 chromosome. Altogether, there are 1,499 TA site hits, located in noncoding regions. Download Table S2, XLSX file, 0.07 MB. Copyright © 2020 Foreman et al. 2020 Foreman et al. This content is distributed under the terms of the Creative Commons Attribution 4.0 International license.

## DISCUSSION

We present a simplified procedure for generating the genomic library for Illumina-based deep sequencing from transposon-mutant pools. The proposed method does not necessitate the design of multiple oligonucleotides and their hybridization and is not dependent on end-to-end ligation. The ease and simplicity of the proposed method does not come at the cost of lower yield—instead, the sequence generated from a library prepared in this method is ∼50% longer, reducing the number of insertion sites that are equivocally mapped (i.e., the number whose location remains undetermined after analysis).

In our opinion, the main advantages of our proposed method are the simplicity and ease of performance, relying on well-known and inexpensive molecular biology techniques. Other Tn-seq techniques have been successfully used for several years now and are definitely of great value. However, we think that use of the proposed technique would be advantageous overall compared to the existing techniques. Compared to the original description of the MmeI-based method (1), our proposal does not necessitate the same use of at least 16 different oligonucleotides (and even more if barcoding is needed), their hybridization, and end-to end ligation of short fragments. Although the method described in reference 1 does not fully mandate running the gDNA digest on a gel and isolating only the fragments that are close to the size of the transposon, this step makes the procedure more specific, as otherwise a vast majority of the heterogeneous primers in the PCR (intended to bind the area next to the MmeI digestion site) would bind nonrelated fragments, making the PCR less specific. In our proposed system, both primers bind inside the transposon, making the reaction more specific even without isolation of the digest by size. Other researchers (3) also proposed modification to the original protocol. In the procedure that they described, they first enriched the junction areas by unidirectional PCR using biotinylated beads, enabling pulldown of the junction-containing fragments specifically. However, that method requires two sequential PCRs, one performed with specialized, bead-affinity primers and the other with the same mixture of multiple primers as that described in reference 1. It is therefore not certain that the method is technically simpler than that described in reference 1.

Our proposed method belongs to the “circularization” methods, as opposed to the “linear PCR” methods. Other circularization methods have been described before. Some involve shearing of the gDNA (mostly by sonication) (8) and then repairing the ends (by a Klenow reactions), self-ligation, and a PCR in which both primers bind inside the transposon (like our proposal). Shearing by sonication is not always simple, and the end repair results depend not only on the presence of an efficient 3′→5′ exonuclease (that needs no dNTPs) but also on that of the much less efficient and dNTP-dependent 5′→3′ polymerase. Another option for self-circularization is the digestion of the genome by one enzyme (or by several, if they all produce compatible ends) that does not digest inside the transposon, followed by self-ligation and PCR from inside the transposon outward. Both these options allow longer stretches of DNA sequences to be identified, which may be an advantage. However, by their nature they produce PCR product that are not of uniform size, ranging from ∼50 to thousands of nucleotides—thus introducing substantial size preference bias during the PCR. Some PCR fragments may even be too long to be synthesized, precluding the identification of the insertion.

There are three other steps in the genomic preparation of the library where bias may theoretically be introduced. The first is the blunting step. If, for example, the ends with CC-3′ are blunted much less efficiently than the ends with AA-3′, then bias may be introduced, as fragments with CC-3′ do not undergo self-ligation. However, we are not aware of such a quality of the blunting reaction/enzyme. In the second step, if the ligase were more effective in ligating blunt ends of one sort or another, this would introduce a similar bias. However, again, we are not aware of such limitations of the T4 DNA ligase that we used. In the third step, all the PCR products from each clone are identical, except the variable 26-bp stretch in the middle. If that stretch is highly problematic for a PCR (unusual secondary structures, highly repetitive sequence, etc.), then the clone may be underrepresented. Such bias would also appear in the “previous” method, as a partially similar stretch needs to be amplified. Additionally, the chances of having such a PCR-unfavorable sequence, considering the modern PCR enzymes available, appear to us to be extremely small.

Using our method, we were eventually unable to match a substantial number of the 26-bp sequence reads generated in the sequencing process to the genome. This was due to mismatch reads introduced during either the outward-looking PCR (producing the 400-bp fragment for the sequencing) or the Illumina procedure itself. For amplification, we used a robust but intermediate-fidelity polymerase. The number of mismatches could potentially be reduced by using a high-fidelity enzyme, as well as by designing the primers to bind at locations closer to (but not directly to) the IR (thus shortening the product and improving the reads). Another factor is that we performed 30 PCR cycles, whereas some resources suggest using fewer (between 12 and 16 cycles). However, these “wrong” sequences are not a source of “false-positive” reads, as we had an eventual “demand” that every mapped read should have a counterpart read from the second direction (every read in R1 mapped to the genome has to appear in R2 and has to map to the complementary strain). In a definitive trial of the system performed on a highly saturated library in M. abscessus, two “runs” consisting of 4.5 million reads each were sufficient to identify over 8,000 Tn mutants, despite what may appear as a low percentage of mapping.

Overall, from the moment one has high quality gDNA until the final 400-bp-long PCR product is ready to be sent for deep sequencing, the procedure can be completed in just under 6 h (assuming a 1-h ligation).

Our Tn-mutant library in M. abscessus is the most saturated library created to date, and the genetic data are presented in a comprehensive way. The analysis of this library, using the presented method, was undertaken in two “steps” of 48 h each, excluding the deep sequencing itself (performed by a commercial company).

In summary, we propose here a technique that is simple to perform—and simple to understand—for the preparation of the genomic library of Himar-1 transposons. Moreover, this simplicity does not come at the cost of lesser performance—in contrast, the results obtained are better (in terms of length of sequence and hence of mapping to genome) than previously published methods. We thus think this that method will both make Tn-seq simpler for those who already know how to do it and make it accessible for the large fractions of biologists who were discouraged by the apparent complexity of, and the preparations needed for, the previous methods.

## MATERIALS AND METHODS

To compare the performance of the “present” system to that of the proposed one, calculating the number of unique compared to ambiguous “hits” for an N_14_-plus-TA system or an N_12_-TA-N_12_ system was done by the use of a code that can be found at https://github.com/MarkBio/In-silico-Analysis-Transposon-Sequencing.

### Creation of the genomic library in M. abscessus transposon mutants.

Transposition of M. abscessus ATCC 19977 was done using a mycobacteriophage with a newly designed transposon that carries a zeocin selection marker. Bacteria were plated on 7H10 plates with 50 μg/ml zeocin. For the “limited,” R→S transition analysis, 200 rough-appearing colonies were pooled and grown overnight in 7H9/oleic acid-albumin-dextrose-catalase (OADC)/glycerol/Tween media. High-quality gDNA was extracted using lysozyme and phenol-chloroform-isoamyl alcohol (IAA). DNAs (15 μg) were digested by the use of 30 units of MmeI (NEB [New England Biolabs]) in a total volume of 300 μl in the presence of 32 mM SAM (S-adenosyl methionine) for 2 h at 37°C. After purification on a column (Macherey-Nagel; Nocleospin [reference 740609.250]), half of the digested DNA was blunted using T4 DNA polymerase (NEB) at 12°C for 15 min. The mixture was cleaned on a similar column and eluted in 50 μl H_2_O. T4 DNA ligase (NEB) was added to all the elute volume in the appropriate buffer, and the ligation reaction mixture was left overnight at room temperature and was finally purified on a column into 50 μl H_2_O. Of that elution, a 2-μl volume was used as the template in a PCR with Illumina-compatible primers, using GoTaq Green master mix polymerase (Promega) (30 PCR cycles; elongation for 30 s; annealing temperature, 63°C).

For the analysis of the full library, the same procedure was repeated, but with pooling of all zeocin-resistant colonies.

### Parallel sequencing on an Illumina platform.

Parallel sequencing was performed at the next-generation sequencing (NGS) facility of Hy-Laboratories Ltd. Per the procedure of the sequencing facility, an additional PCR step consisting of 10 cycles was performed on each sample. However, researchers performing their own deep sequencing may forgo this step, as long as the primary PCR was performed with Illumina-compatible primers. Products were checked by Qubit and by Tapestation. Sequencing was done in Illumina MiSeq sequencer, using a MiSeq V2 kit for 500 cycles to generate 2 × 250 paired-end reads.

### Extracting the actual genetic sequence from the mass-sequencing data and mapping to the M. abscessus genome.

As noted above, the code details can be found at https://github.com/MarkBio/In-silico-Analysis-Transposon-Sequencing. Raw data from the sequencing can be found at that link as well, through a Dropbox link.

### Data availability.

Accession numbers for the sequences of the 30 bacterial organisms that were analyzed *in silico* are listed in Table 1.
